# Liposome-Tethered Gold Nanoparticles Triggered by Pulsed NIR Light for Rapid Liposome Contents Release and Endosome Escape

**DOI:** 10.3390/pharmaceutics14040701

**Published:** 2022-03-25

**Authors:** Anisha Veeren, Maria O. Ogunyankin, Jeong Eun Shin, Joseph A. Zasadzinski

**Affiliations:** 1 Chemical Engineering and Materials Science, University of Minnesota, Minneapolis, MN 55455, USA; aveeren@umn.edu (A.V.); mariaolu.ogunyankin@bms.com (M.O.O.); shinx372@umn.edu (J.E.S.); 2 Bristol Myers Squibb, 1 Squibb Drive, New Brunswick, NJ 08902, USA

**Keywords:** nanobubble, plasmon-resonant, hollow gold nanoshell, picosecond laser pulses

## Abstract

Remote triggering of contents release with micron spatial and sub-second temporal resolution has been a long-time goal of medical and technical applications of liposomes. Liposomes can sequester a variety of bioactive water-soluble ions, ligands and enzymes, and oligonucleotides. The bilayer that separates the liposome interior from the exterior solution provides a physical barrier to contents release and degradation. Tethering plasmon-resonant, hollow gold nanoshells to the liposomes, or growing gold nanoparticles directly on the liposome exterior, allows liposome contents to be released by nanosecond or shorter pulses of near-infrared light (NIR). Gold nanoshells or nanoparticles strongly adsorb NIR light; cells, tissues, and physiological media are transparent to NIR, allowing penetration depths of millimeters to centimeters. Nano to picosecond pulses of NIR light rapidly heat the gold nanoshells, inducing the formation of vapor nanobubbles, similar to cavitation bubbles. The collapse of the nanobubbles generates mechanical forces that rupture bilayer membranes to rapidly release liposome contents at the preferred location and time. Here, we review the syntheses, characterization, and applications of liposomes coupled to plasmon-resonant gold nanostructures for delivering a variety of biologically important contents in vitro and in vivo with sub-micron spatial control and sub-second temporal control.

## 1. Introduction

Following their discovery by Bangham in 1965 [1,2], liposomes have been one of the most thoroughly investigated nanocarriers for drug delivery [3,4,5,6,7,8,9,10,11,12,13]. Many promising drugs are discarded due to difficulties in maintaining a safe biodistribution in the concentration range necessary for efficacy [14]. Liposomes and other lipid-based drug carriers sequester toxic drugs within a lipid bilayer to alter drug biodistribution, thereby enhancing efficacy while minimizing damage to healthy tissue and organs [14]. Liposomes are capable of concentrating and stabilizing small molecule therapeutic compounds and larger oligonucleotides for delivery in vivo and in vitro [12,15,16,17,18]. Liposomes based on naturally occurring lipids are inherently biocompatible and hence can overcome many of the obstacles to cellular and tissue uptake. Small liposomes (<200 nm) are passively targeted to tumors and sites of inflammation by the enhanced permeation and retention (EPR) effect [18,19]. Liposomes can also be actively targeted by treating their surface with antibodies to ligands overexpressed on tumor cells [20,21,22,23,24,25,26]. Hydrophilic cargoes can be encapsulated within the aqueous liposome core, while hydrophobic molecules can be trapped within the hydrophobic bilayer interior [27].

### 1.1. Separating Cargo Sequestration from Rapid Release

Despite these advantages, it is difficult for a single bilayer to simultaneously prevent drug release and trigger rapid release at the preferred site of action [28]. It remains a challenge to initiate a biological or chemical response in cells or tissues with spatial and temporal control. Conventional liposomes often release small molecule drugs prematurely in the circulation due to degradation of the liposome bilayer or are taken up in the liver and spleen by interactions with the reticuloendothelial system (RES). “Stealth” coatings of polyethylene glycol and other polymer lipids can prolong the circulation time [29,30]. However, optimizing the liposome cargo retention in the circulation to maximize drug accumulation at the disease site may slow drug release from the liposome at the site of interest and reduce therapeutic activity. This is the case for clinical doxorubicin liposomes (Doxil) [31,32,33] and for clinically tested cisplatin liposomes [34]; drug release is so slow that the critical drug concentration is not easily achieved. As a result, multiple strategies for enhancing local drug release at the site of interest have included the incorporation of membrane destabilizing agents that respond to stimuli such as general hyperthermia [35,36,37], receptor targets [38,39,40], pH changes [41], or tumor-specific enzymes [42,43]. These strategies promote release but can compromise long-term liposome stability and decrease drug retention in the circulation. Specific ligands with high affinities to receptor overexpressed on disease cells can in turn lead to “binding-site barriers” where high concentrations of bound nanocarriers can prevent drug penetration into tissue [44]. 

### 1.2. Plasmon-Resonant Gold Nanostructures

**Figure 1:**
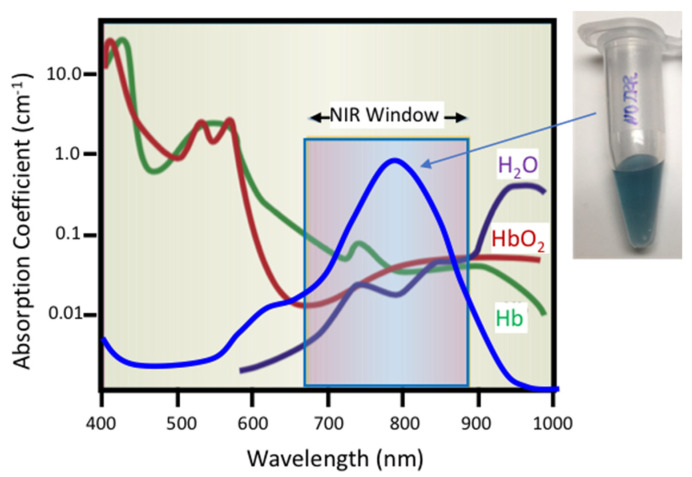
Schematic representation of optical adsorption of water (H_2_O), oxygenated hemoglobin (HbO_2_), and hemoglobin (Hb) showing the near-infrared window (NIR) from 650–900 nm over which physiological materials do not adsorb significant fractions of incident light, adapted from [54], published by Nature, 2001. Hollow gold nanoshells (HGN) have a surface plasmon resonance determined by the HGN wall thickness and diameter that can be tuned to peak in the NIR Window. This causes HGN suspensions to appear dark blue to purple.

To address the challenge of spatially and temporally controlled fast release, modified liposome nanocarriers have been developed in which the liposome functions primarily as the cargo container. Rapid spatial- and temporal-controlled contents release is addressed by tethered gold nanostructures triggered by an externally applied pulsed NIR laser. Such liposomes are also well-suited for in vitro, in vivo, and ex vivo use where spatial- and temporal-controlled delivery of cargo ranges from small molecules to complex proteins or oligonucleotides [45,46,47,48]. Plasmon-resonant hollow gold nanoshells (HGN) or simple gold nanoparticles [46,47,49,50] can be chemically tethered to conventional polyethylene glycol stabilized liposomes of any membrane composition to absorb and concentrate near-infrared light (NIR) [32,33,48,51,52,53]. A hollow shell structure of gold, silver, or alloys can strongly absorb NIR light at a characteristic localized surface plasmon resonance (LSPR) wavelength (Figure 1) that can be tuned by controlling the nanoparticle size and shell thickness [45]. As shown schematically in Figure 1, the absorption spectra of common physiological molecules such as water, oxygenated hemoglobin (HbO_2_), and hemoglobin (Hb) show a distinct minima in what has been called the “NIR window” [54]. As a result, NIR light can penetrate deeply within cell suspensions, tissue, and other physiological media. Unstructured solid silver or gold nanoparticles of 10–100 nm size absorb in the same wavelength range as hemoglobin (400–540 nm) and cannot be readily addressed in physiological fluids.

**Figure 2:**
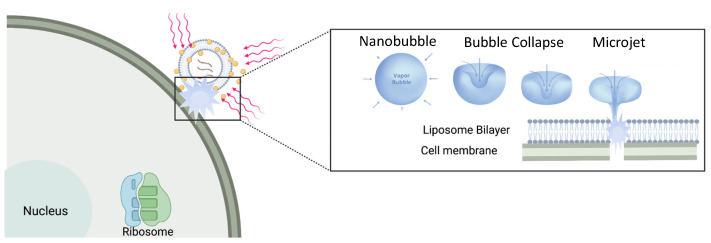
Plasmon-resonant gold nanoparticles tethered to liposomes can be triggered by short pulses of NIR laser light (red lines) that heat the nanoparticles. At a threshold light fluence, heat dissipating from the nanoparticles boils a minute amount of water to form unstable vapor nanobubbles that rapidly expand and contract. As the nanobubbles collapse, liquid–vapor microjets form that can perforate cell and liposome membranes. This allows the liposome contents to be rapidly released and cells to be perforated.

By manipulating the shape and organization of gold nanostructures, the surface plasmon-resonance can be moved to the NIR window. Light absorption from high power nanosecond or shorter pulses cause the gold nanostructures to undergo a rapid increase in temperature before any heat can be dissipated to the surrounding fluids [45,55]. Following this rapid heating of the nanoparticle, the hot nanoparticles begin to dissipate their thermal energy to the surrounding solution in microseconds. The high temperature gradients around the gold nanoparticles vaporize a minute amount of the surrounding water leading to the formation of unstable nanobubbles as the heat is dissipated (30 nm diameter particles thermally equilibrate with their surroundings in fractions of a microsecond). These nanobubbles rapidly expand and contract and give rise to microjets that have similar mechanical and thermal effects as ultrasound-induced cavitation (Figure 2) [51,56,57]. Nanobubble generation requires a threshold energy; any additional energy above threshold makes the nanobubbles grow larger. Once formed, the mechanical forces generated by the expansion and collapse of the nanobubbles can lyse endosomes, liposomes, or cells, providing rapid contents release [51,58,59,60,61], cell poration [62,63], or even cell death [62,63,64,65]. It is these mechanical cavitation forces that lead to endosome, liposome, and cell membrane rupture and contents release rather than the change in temperature. As a result, complex proteins and oligonucleotides, as well as small molecules, can be delivered without damage following NIR light triggering of the gold nanostructures [47,48,58,61,66].

### 1.3. Summary

Here we review the synthesis and characterization of a variety of plasmon-resonant gold nanostructures that show strong absorption in the NIR window that can be tethered to, encapsulated within, or grown directly on conventional liposome carriers. Gold nanoparticles are stable and non-toxic in physiological environments and can be synthesized in various structures and shapes including nanoshells, nanospheres, nanocubes, nanowires, nanocages, nanoflowers, nanopyramids, and nanobranches [67,68]. Nanobubbles can be triggered around these gold nanostructures using focused near-infrared laser light pulses to spatially and temporally control release of small molecules, siRNA, and DNA within cells, in cell suspensions, or in tissue to initiate various biophysical or biochemical responses. Gold nanoparticles can be bound directly to the small molecule, protein [61,66,69], or oligonucleotide of interest [58,59,60,70]; however, this review focuses on gold nanoparticles directly bound to conventional liposomes to trigger rapid, localized small molecule or oligonucleotide delivery [32,33,45,46,47,48,49,50,51,52,53]. Gold nanoparticles and liposomes show minimal toxicity; the unique plasmon resonance properties of gold nanostructures combined with the simple synthetic methods needed to alter this plasmon adsorption, the ease of gold surface functionalization and binding to liposome membranes, and the ability of liposomes to sequester and protect almost any water-soluble cargo combine to create a uniquely useful nanocarrier.

## 2. Plasmon-Resonant Gold Nanoparticles

To create a nanoparticle with a localized surface plasmon resonance (LSPR) in the near-infrared region of the spectrum requires a low dielectric constant core such as water [45,71], silica [72,73,74,75,76,77,78], lipid bilayers [47,49,50,79], or polymers [80,81], with a thin shell of high dielectric gold or a gold–silver alloy, for use in aqueous or physiological solutions. Such particles are known as “hollow gold nanoshells” or HGN. The typical techniques used to create core-shell HGN nanostructures are sacrificial galvanic replacement of cobalt [82] or silver nanoparticle templates [45,48,51,55,71,83,84,85], or using a silica [73,74,75,76,77,86], polymer, or liposome [46,47,49,50] core onto which the gold nanoparticles precipitate. The plasmon resonance depends on the ratio of the thickness of the dielectric shell to the dimensions of the core that allows the LSPR maximum, $\lambda_{max}$, to be shifted from the 400–500 nm for solid gold or silver nanoparticles to >1000 nm.

### 2.1. Synthesis of HGNs via Galvanic Replacement of Cobalt Nanoparticles

**Figure 3:**
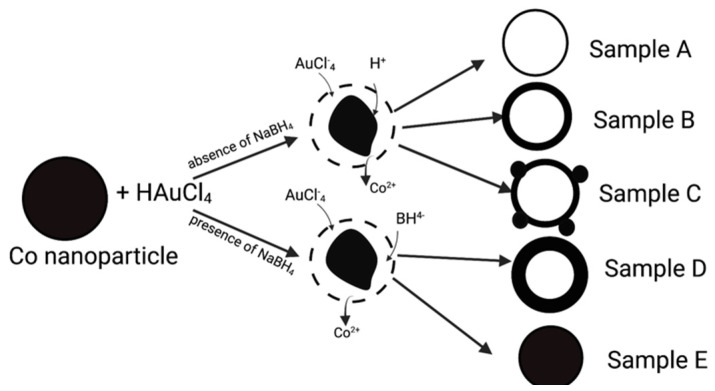
Synthesis of hollow gold nanoshells from cobalt templates. Any residual NaBH_4_ from the cobalt template reaction can lead to additional uncontrolled gold deposition resulting in polydispersity of the nanoshell size and wall thickness. (Adapted from [82], published by American Chemical Society, 2005).

**Figure 4:**
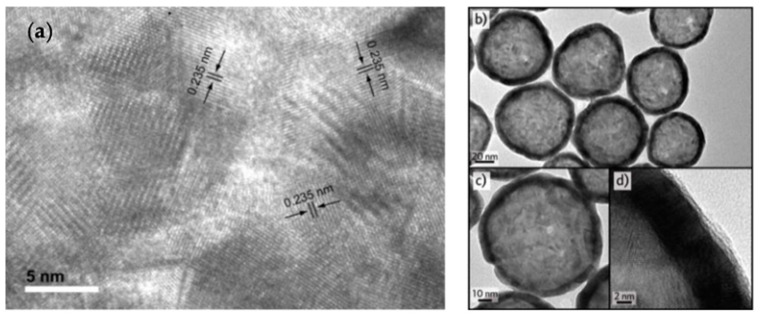
High resolution TEM images of hollow gold nanoshells templated from cobalt nanoparticle templates. (**a**) Lattice image of gold nanoshell showing the characteristic 0.235 nm lattice spacings of crystalline gold. Reprinted with permission from ref. [82], Copyright 2005 American Chemical Society. (**b**) Collection of cobalt-templated HGN showing spherical shapes with dark rims. (**c**) Higher magnification image of nanoshell. The dark rim and lighter interior is consistent with a gold shell structure. (**d**) Close-up of gold shell showing uniform shell thickness.

**Figure 5:**
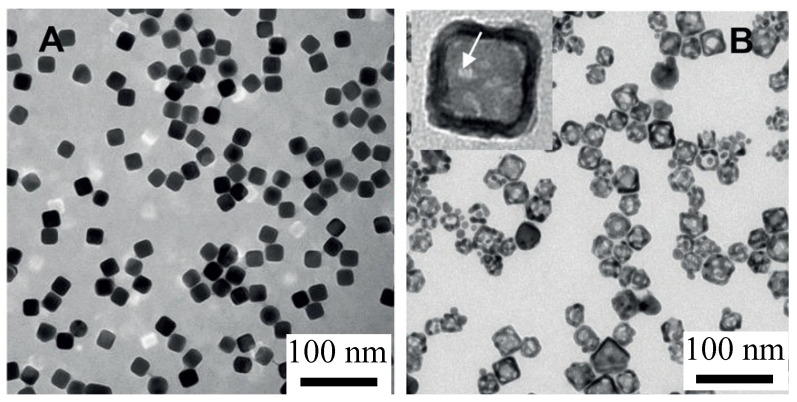
(**A**) Transmission electron micrograph of silver nanocubes formed by the polyol reaction following 30 min of growth. The cubes are monodisperse with well-defined sharp edges. The mean size of the silver nanocubes is 18 nm. (**B**) TEM image of hollow gold nanoshells (HGN) made from the silver templates in (**A**) by the galvanic replacement according to the reaction in Equation (3). The inset shows a high magnification image illustrating the hollow shape with pores in the walls (arrow). The mean size of the hollow gold nanocubes in (**B**) increased to 23 nm, and the size distribution broadens. Adapted from [45], published by Wiley, 2018.

Liang et al. [82] first reported on the sacrificial galvanic replacement of cobalt nanoparticles based on the Kobayashi method for hollow platinum nanoshell synthesis (Figure 3 and Figure 4). In all these sacrificial processes, a metal nanoparticle template is synthesized, followed by a galvanic replacement reaction with a gold salt. The template nanoparticles must have a lower redox potential than gold, which causes the spontaneous reduction and plating of gold onto the dissolving oxidized template. Cobalt (Co^2+^/Co 0.3V, vs. SHE) or silver (Ag^+^/Ag 0.8V, vs. SHE) templates have lower redox potentials than gold (AuCl_4_^−^/Au 0.99V, vs. SHE) [45,71,82,87]. During galvanic replacement, the gold salt is reduced to (Figure 4 and Figure 5) metal on the template and retains the shape and size of the template. The oxidized template metal dissolves into the solution, leaving behind a hollow core (Figure 4 and Figure 5). The template size and polydispersity directly control the size and polydispersity of the final hollow gold nanoparticles [45]. For the cobalt templates, an excess amount of the reducing agent NaBH_4_ was used as reducing agent for CoCl_2_ in degassed water under constant nitrogen flow, which led to cobalt nanosphere formation (Equation (1)).

(1) $$2{\mathrm{NaBH}}_{4}+{\;\mathrm{CoCl}}_{2}\;\to {\;\mathrm{Co}}^{\left(0\right)}+{\;H}_{2}O\;+2\mathrm{NaCl}\;+B_{2}H_{6}$$

After completion of the reaction, the cobalt nanosphere solution is washed and added to a solution of HAuCl_4_, which leads to the oxidation of the cobalt, and the gold chlorate is reduced to gold metal that precipitates onto the template by Equation (2).

(2) $$3\mathrm{Co}\;+2{\mathrm{AuCl}}_{4}^{-}\;\to \;3{\mathrm{Co}}^{2+}+2{\mathrm{Au}}^{\left(0\right)}+8{\mathrm{Cl}}^{-}$$

The cobalt nanosphere acts as the primary reducing agent in Equation (2), which is why it is important to remove any excess NaBH_4_ from the solution. Any remainingNaBH_4_ can cause direct gold reduction and thicker gold nanoshells to be formed, and it can prevent the cobalt core from being oxidized, which increases the polydispersity of the core diameter and shell thickness (Figure 3). As the nanoshell size and shell thickness control the local surface plasmon resonance [45], the better controlled the reaction conditions, the more precisely the LSPR can be located. It is also necessary to remove dissolved oxygen from the solution by purging with nitrogen or argon to reduce premature oxidation caused by ambient conditions to avoid extraneous oxidation reactions. The high reactivity of cobalt makes the syntheses more complicated than silver as described below. A second drawback is that cobalt toxicity has been observed following chronic exposure in patients with metal-on-metal hip replacements that use cobalt, although it is unlikely that the amount of residual cobalt in these nanoparticles would be problematic [88,89].

### 2.2. Synthesis of HGNs via Galvanic Replacement of Silver Nanoparticles

The use of silver nanoparticle templates for HGN synthesis was introduced by Sun et al. [71]. The lower reactivity of silver relative to cobalt provides a simpler and more readily reproducible synthetic method for gold nanoshell formation [45,87]. Silver toxicity is considered quite low. Silver is an important part of dental amalgams, and silver and silver nitrate compounds are used as antimicrobials [90].

Silver templates can be made with precise control of the nanoparticle size and shape (Figure 5A) using the polyol process first described by Xia and coworkers [71,83]. Cubic silver templates are synthesized by reduction of silver trifluoroacetate in diethylene glycol in the presence of polyvinyl pyrrolidone heated to 150 °C (Figure 5A) [87,91]. The synthesis is initiated by a rapid nucleation of silver crystals that is indicated by a color change to pale yellow. Following nucleation, there is a slow growth of the silver templates as cubic crystals stabilized by the polyvinyl pyrrolidone [87]. Figure 5A shows that the silver templates are monodisperse in shape and size; cubic silver nanocrystals can be made with edge lengths from 10 to 50 nm through this one-pot process [45]. To minimize aggregation and settling during silver nanoparticle storage, sodium citrate is added. The citrate binds to the silver templates and provides an electrostatic barrier to aggregation and sedimentation.

**Figure 6:**
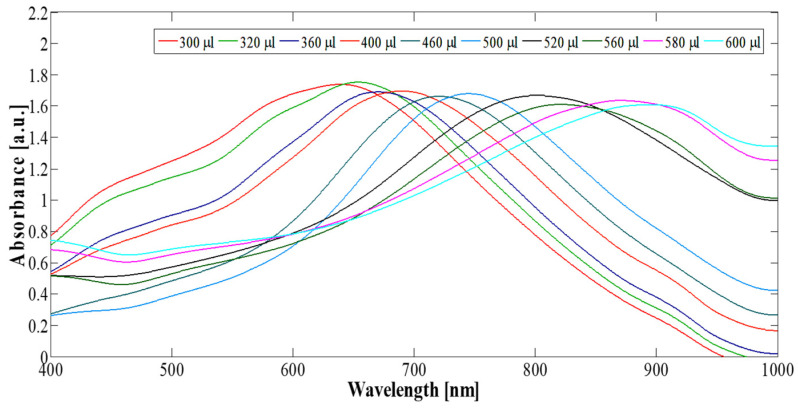
Adding increasing amounts of 1 mM HAuCl_4_ to a fixed amount of 27 nm cubic silver template solution redshifts $\lambda_{max}$ from 600 to 900 nm. Increasing the amount of gold thins the HGN shell leading to the increase in $\lambda_{max}$. Adapted from [45], published by Wiley, 2018.

Figure 6 shows that the wavelength, $\lambda_{max}$, at which the LSPR is a maximum is determined by a combination of the size distribution of the silver nanoparticles used as sacrificial templates and the ratio of gold salt to silver in the subsequent galvanic replacement reaction [45,71]. HGN can be made with a variety of shell thicknesses using Turkevich’s colloidal growth chemistry to oxidize and dissolve the sacrificial silver nanoparticles while metallic gold is plated on the template exterior [92].

(3) $${\mathrm{Ag}}_{\left(s\right)}+{\mathrm{AuCl}}_{4\left(\mathrm{aq}\right)}^{-}\to {\mathrm{Au}}_{\left(s\right)}+3{\mathrm{Ag}}_{\left(\mathrm{aq}\right)}^{+}+4{\mathrm{Cl}}_{\left(\mathrm{aq}\right)}^{-}$$

As the reaction proceeds, the solution color changes from yellow to blue (Figure 1), consistent with the red-shift of the localized surface plasmon resonance (LSPR) from 420 nm to 700–950 nm, depending on the ratio of gold chlorate salt to silver (Figure 6) [45]. As more gold is added, more silver is oxidized, and the shell thickness actually decreases as three silver molecules are oxidized for every gold molecule reduced to metal. During this process of oxidation and reduction, the gold metal mixes with any remaining metallic silver from the template to form a gold/silver alloy in the walls of the HGN [45,87,93].

To stabilize the final HGN against aggregation in physiological saline solutions, 750, 2000, or 5000 Da methoxy-PEG-thiol can be coupled to the HGN surface. The excess PEG can be removed by repeated washing and centrifugation. PEG-stabilized HGN are extremely stable in saline and serum, and the spectra and nanostructure have remained unchanged for more than a year. HGN made by other silver templated syntheses may be less stable [94].

### 2.3. Synthesis of Gold Shells on Modified Silica Nanoparticles

**Figure 7:**
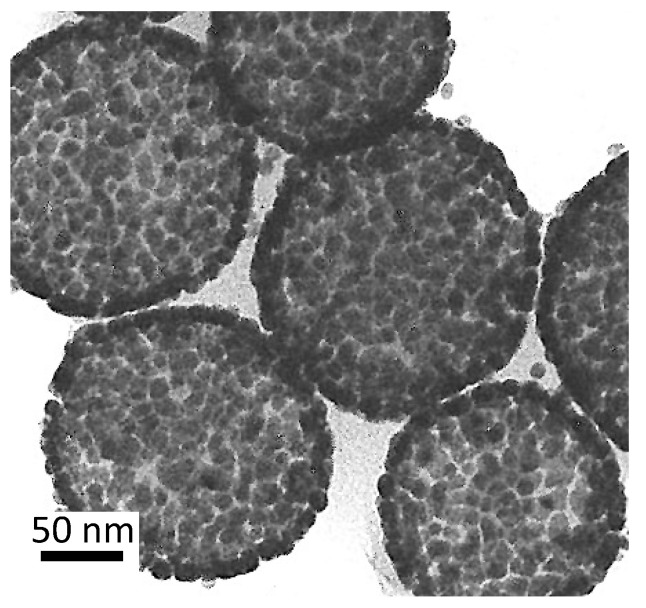
TEM image of nanoshells prepared by seeding gold nanoparticles onto a silica core, followed by a second reduction of gold chlorate to create a continuous gold film. Adapted from [78], published by ACS, 2005.

Halas and coworkers synthesized cores of monodisperse silica particles, followed by binding gold nanoparticle seeds to the silica using coupling agents. The monodisperse silica spheres are prepared by the Stöber method [95], then coated with 3-amiopropyl triethoxysilane [96]. The 1–3 nm gold nanoparticles are synthesized by the technique outlined by Duff [97] and mixed with the silica solution. The gold particles react with the amine groups on the organosilane and bind to the surface of the silica sphere. These gold nanoparticles act as nucleation sites for the reduction of chloroauric acid to gold with formaldehyde. The gold particles on the surface of the particles grow and can coalesce to form a continuous shell around the silica sphere (Figure 7). The density of the gold nanoparticles and the space between nanoparticles on the surface of SiO_2_ can be tuned to provide control the amplitude and $\lambda_{max}$ of the LSPR [78,98]. 

### 2.4. Synthesis of Gold Shells on Lipid Bilayer Liposomes

Romanowski and coworkers introduced a method for directly precipitating gold nanoparticles and binding them to liposomes [49,50]. The gold coating provides a tunable LSPR while maintaining the carrier properties of conventional liposomes. The gold precipitates as discrete clusters on the liposome surface and can eventually grow into a continuous metallic shell [46,47]. As in other core-shell structures, the $\lambda_{max}$ of the LSPR can be varied from 600 to 800 nm. The magnitude and wavelength of the LSPR is dependent on the quantity of the gold reduced, as is the case for the gold-coated silica spheres (Figure 7) [46,47,49,50]. 

**Figure 8:**
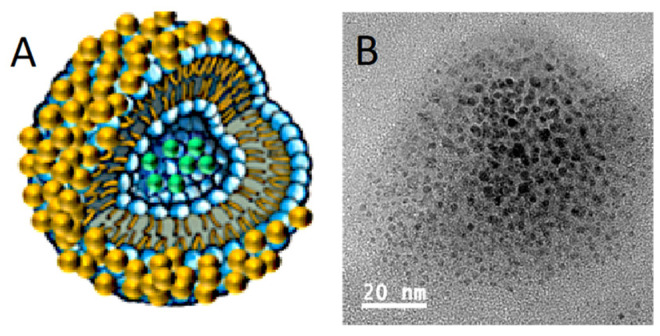
(**A**) Schematic diagram of gold nanoparticles nucleating on the liposome surface. (**B**) TEM image of liposome decorated with gold nanoparticles. Adapted from [46,47], published by Wiley, 2017 and Wiley, 2020.

Gold nanoparticles are nucleated onto the liposome surface by adding 10 mM gold chloride solution to a liposome suspension (0.66 mM lipid concentration) at a molar ratio of 1:2 gold to lipid followed by the addition of an equal volume of 40 mM ascorbic acid solution until a characteristic color develops [47]. Following reduction, the samples are dialyzed against the appropriate buffer at room temperature to remove unreacted gold chloride and ascorbic acid. The resulting structures are shown schematically and in the TEM image in Figure 8 [47].

### 2.5. How HGN Geometry, Size, and Wall Thickness Determine the LSPR

The amplitude of the adsorption cross section, *σ_abs_,* as a function of wavelength for core-shell nanoparticles can be approximated from Mie theory for core-shell spheres [45]. This analytical solution for *σ_abs_* for spherical hollow metal nanoshells with particle diameters much smaller than the wavelength of the incident light [99] gives insight into how the composition, shape, and size of the nanoshell determine the LSPR. The nanoshell is characterized by a total radius, *R*, (core radius + shell thickness) and a shell thickness, *t*. For typical hollow gold nanoshells (Figure 5B), the ratio of shell thickness, *t*, to overall size, *R,* varies from 0.10 < *t/R* < 0.25 [45,99]. Water, with a constant real permittivity of $\epsilon_{W}=1.77,$ serves as both the core of the hollow shells and the external medium. For the HGN synthesis described previously, the shell is made up of a gold–silver alloy with dielectric function $\epsilon_{G}\left(\lambda \right)$, which has real and imaginary (Im) frequency-dependent parts. For a Ag fraction of $x_{Ag}$, the complex permittivity of the gold–silver alloy is [100]

(4) $$\epsilon_{G}\left(\omega \right)=\left(8.6-4.6\times x_{Ag}\right)-\frac{{\left(8.96+0.02x_{Ag}\right)}^{2}}{\omega^{2}+i\omega \left(0.06+0.47x_{Ag}-0.46x_{Ag}^{2}\right)}.\;\;\;\;\;\;\;\;\;\;\;\;$$

For $\frac{t}{R}\ll 1$, *σ_abs_* for a spherical shell is given by the approximate expression [99]

(5) $$\sigma_{abs}\approx \left(R^{2}t\right)\left[\frac{8\pi^{2}\sqrt{\epsilon_{W}}}{\lambda }\right]Im\left(\frac{\left(2\epsilon_{G}{}^{2}-\epsilon_{W}\epsilon_{G}-\epsilon_{W}{}^{2}\right)}{3\epsilon_{G}\epsilon_{W}}\right)\;\sim \;\xi R^{2}t.\;\;\;$$

This shows that *σ_abs_~ξR^2^t*, in which *ξ* is a frequency-dependent function of the dielectric constants of water and metal, and *R*^2^*t* are proportional to the volume of the metal in the nanoshell. 

**Figure 9:**
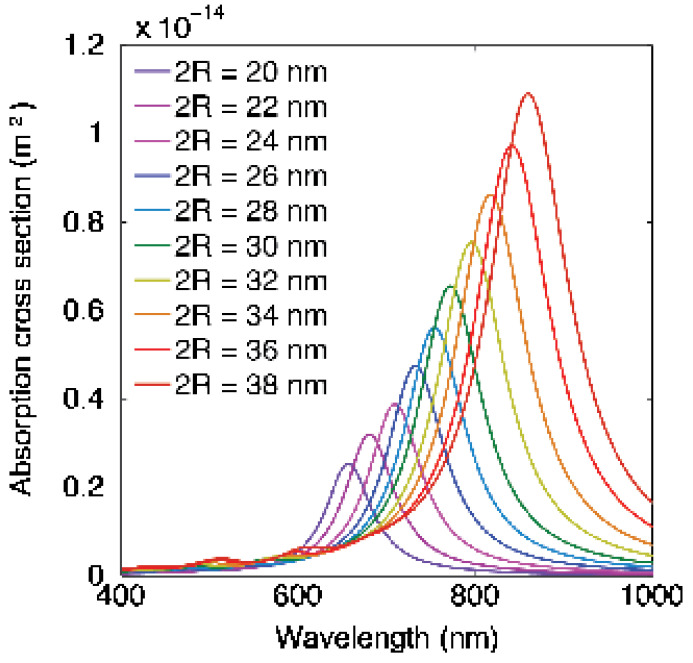
Full-field electromagnetic simulations of 50% Ag–50% Au alloy cuboidal HGN absorption cross section for a HGN wall thickness of 2 nm for various edge lengths. Decreasing the ratio of wall thickness to edge length red-shifts the spectra. Increasing the edge length increases the magnitude of the adsorption cross section as ~$R^{2}t$, as suggested by the analytic Mie theory (Equation (5)). Adapted from [45], published by Wiley, 2018.

Figure 9 shows *σ_abs_* calculated from full-field finite-difference time-domain (FDTD) electromagnetic simulations performed using numerical FDTD algorithms for cube-shaped HGN (Figure 5) [101]. The shell geometry red-shifts $\lambda_{max}$ of the LSPR into the NIR window. Increasing the edge length increases the magnitude of *σ_abs_* as $R^{2}t$, as suggested by the simple Mie theory (Equation (5)) [45]. The sharper edges of the cuboids also increase the adsorption cross section compared with the spherical geometry [45], while the gold–silver alloy composition of the shells blue-shifts the *σ_abs_* [45]. The experimental *σ_abs_* (Figure 6) are typically much broader than the calculated *σ_abs_* in Figure 9. Figure 5B shows that there is polydispersity in shape, size, and wall thickness of the synthesized HGN, while Figure 9 shows the sensitivity of the calculated *σ_abs_* to small changes in the HGN geometry, which allows for significant tunability of the optical properties. However, any polydispersity in shape, size, and/or wall thickness broadens the experimental *σ_abs_*, as seen in Figure 6.

### 2.6. Localized Heating via HGN Absorption of Continuous NIR Light

There are two biological windows for NIR light: the first, from 650 to 900 nm (NIR-i) and the second at 1000 to 1350 nm (NIR-ii). While the NIR-ii window may provide better tissue transmission via lower scattering and lower background absorbance, it is difficult to synthesize nanoshells that are responsive in the NIR-ii window using current synthetic pathways. Figure 9 shows that moving the LSPR to 1000 nm or more would require large nanoshells or small wall thicknesses, which would not penetrate tissues as well or be easily coupled to liposomes. Therefore, the use of nanoshells in the NIR-ii window has not been thoroughly studied.

Photothermal energy conversion using either continuous or pulsed laser irradiation can be used to locally heat microscale volumes with plasmon-resonant gold nanoparticles without heating the bulk of the solution. Heating with continuous laser irradiation is commonly referred to as photothermal heating [73,102,103], and can be used to induce drug release from polymers [104,105,106], or localized hyperthermia to kill cancer or bacterial cells [73,74,107,108,109,110], or to release drugs from temperature sensitive liposomes [32,33,53]. NIR heating of gold nanoshells has also been used to denature proteins used in “stitchless” wound closure [111].

However, continuous laser irradiation of gold nanoparticles or gold/silica nanoshells only generates small, steady-state temperature increases in the nanoparticles above the average local solution temperature (Δ*T* ≈ 1–4 °C) [33]. The ratio of nanoparticle surface area to volume leads to equilibration times of microseconds, so the nanoparticle temperature when continuously irradiated with low power light is always close to the surroundings temperature [32,33,53]. Hence, for continuous irradiation the nanoparticles act as a local energy source for heating the bulk solution. The magnitude of the local heating depends on the local nanoparticle concentration, the laser intensity, and the length of irradiation. A uniform, elevated temperature distribution in a specific volume, such as a tumor, is difficult to control due to the variable distribution of nanoparticles throughout the volume as well as the often quite different thermal and transport properties of different tissues, blood, and bone, as well as the natural convective losses due to blood and lymphatic fluid flow. The temperature rise in off-target tissues must be limited to prevent thermal damage as well as off-target drug release. However, under optimal conditions, the local tissue temperature can be raised to 45–50 °C, sufficient to denature proteins and kill tumor and other types of cells [112,113]. A recent clinical trial has shown that thermal ablation of prostate cancer tumors is possible using continuous laser irradiation of silica–gold nanoshells (Figure 7) [112]. Use of continuous, low power NIR-induced heating using gold and other plasmon-resonant nanoparticles is reviewed in [114] but is not the focus here.

### 2.7. Localized Nanobubble Formation via HGN Adsorption of Pulsed NIR Light

The rate and power at which the NIR light is delivered to the gold nanoparticles dictates the thermal and mechanical effects of NIR irradiation. Nanosecond and shorter laser pulses can deliver optical energy to gold nanostructures faster than the thermal energy can dissipate to the external surroundings [55,115,116,117], unlike the continuous low power irradiation discussed in the previous section. The characteristic time for thermal dissipation is τD~ ρCpR2k ; $\rho C_{p}$ is the volumetric heat capacity of gold (2.5 × 10^6^ J·m^−3^·K^−1^), *k* is the thermal conductivity of water (0.6 W·m^−1^·K^−1^), and *R* is the nanoparticle radius. For 5 ≤ *R* ≤ 50 nm, 100 ≤ $\tau_{D}$ ≤ 10,500 psec. This likely underestimates the characteristic time because at the nanoscale, an energy mismatch of the vibrational modes at the metal–water interface can limit heat transfer. Heat transfer may be further complicated by thiol-bound PEGs or other ligands of various molecular weights. This leads to an interfacial resistance to heat transfer that can increase $\tau_{D}$ [118]. For laser pulse lengths less than $\tau_{D}$, the adsorbed light energy is confined to heating the nanoparticle; hence, the temperature increase is directly proportional to the light energy adsorbed (Equation (6)) [45,115]. The temperature increase in the HGN can be sufficient to melt the hollow gold shape, causing a change in the HGN shape, which in turn, alters the LSPR. 

The absorption cross section is proportional to the metal shell volume, $\sigma_{abs}≅\xi R^{2}t$, (Equation (5)), and the energy absorbed is $Q=\sigma_{abs}F≅\xi R^{2}tF,\;$in which *F* is the laser fluence (energy/area). To generate a nanoscale bubble, the water must be superheated to overcome the effects of the surface tension, *γ*, at the bubble–water interface. The required superheating is significant: to nucleate a 10 nm radius bubble requires superheating water to at least 272 °C [119]. However, at this temperature, water has essentially reached its spinodal temperature, *T_S_* = 277 °C, at which the liquid becomes unstable and spontaneously converts to the vapor phase with no heat of vaporization [117]. Recent theoretical simulations suggest that the pressure increase inside rapidly heated bubbles reaches the critical pressure as well [118]. 

**Figure 10:**
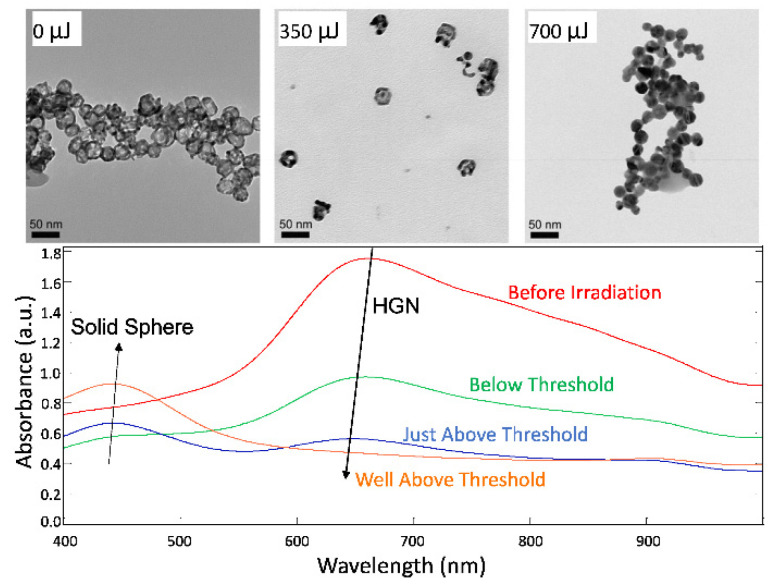
(**top**) TEM images of HGN following 0, 350, and 700 µJ pulse energy irradiation. The HGN evolve from hollow spherical shells to solid spheres following irradiation sufficient to induce nanobubbles. Adapted from [55], published by Wiley, 2008. (**bottom**) Optical spectra of HGN showing $\lambda_{max}$ of the LSPR peak at 700 nm decreases with increasing laser irradiation until this peak disappears well above the threshold laser intensity to induce nanobubble formation. A second LSPR grows in at $\lambda_{max}$ ~450 nm with increasing laser intensity, corresponding to the plasmon resonance of solid silver–gold alloy spheres. Adapted from [45], published by Wiley, 2018.

Hence, to generate a nanobubble, the shell must be heated well above 277 °C. Rapid conversion of the optical energy to heat causes the gold shell temperature to increase to *T_G_*, determined by the light intensity and the HGN adsorption cross section (Figure 9). Subsequently, heat begins to flow to the water in the core of the nanoshell, initially at *T*_0_, causing the water temperature to rise and the shell temperature to fall until they are equalized. Hence the approximate maximum temperature that can be reached by the water in the core is

(6) $$T_{max}=\frac{\left(\frac{t}{R}\right)3\rho C_{p}T_{G}+\rho_{W}C_{pW}T_{0}}{\left(\frac{t}{R}\right)3\rho C_{p}+\rho_{W}C_{pW}}$$

where $\rho_{W}C_{pW}$ (4.2 J·cm^−3^·K^−1^) is the volumetric heat capacity of water. The maximum water temperature depends on *t*/*R* (Figure 6) [99]. For *R* = 10 nm and *t* = 2 nm, for *T_max_* to reach the spinodal temperature of 277 °C, the gold–silver alloy shell must be heated to ~1000 °C, which is close to the melting point of ~1050 °C. The nanoshell also loses heat to the surroundings, which means the metal shell must reach an even higher temperature. TEM images (Figure 10, top) show that the hollow gold structure collapses following nanobubble formation, confirming the intense temperature increase. The optical spectra (Figure 10, bottom) shows that the LSPR peak at ~700 nm decreases with increasing irradiation intensity, while the LSPR for solid, spherical silver–gold nanoparticles at ~450 nm increases, consistent with reaching high enough temperatures that cause the hollow shells to melt into solid spheres [45,55].

**Figure 11:**
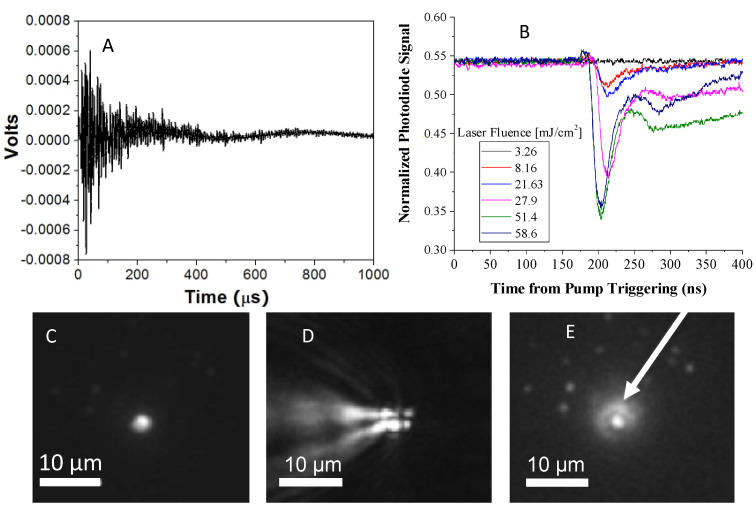
(**A**) Hydrophone voltage variations corresponding to pressure changes in a solution of HGN following irradiation with pulsed NIR light. The pressure variations correspond to the formation and collapse of nanobubbles, similar to the pressure variations induced by cavitation bubbles during sonication. Figure adapted from [51]. (**B**) The decrease in photodiode signal of a continuous laser probe beam is due to the scattering from the nanobubbles and increases with increasing laser fluence consistent with larger or more numerous nanobubbles. A fluence of ~3 mJ/cm^2^ is not sufficient to induce nanobubbles, and there is no change in the probe signal (black trace). Higher fluences show the decrease in signal with a threshold fluence of ~8 mJ/cm^2^ for nanobubble formation (red trace). Adapted from [45], published by Wiley, 2018. (**C**) Optical microscope image of fluorescently labelled liposome tethered to a glass coverslip. (**D**) Ten nanoseconds after pulsed NIR laser showing the formation of a liquid–vapor jet due to the rapid expansion and collapse of the nanobubble. The jet induces mechanical stress to the liposome membrane as in sonication, as in Figure 2. Bar is 10 µm. (**E**) Fluorescent dye is released from the liposome in C after 10 msec (arrow). Adapted with permission from [8], published by Elsevier, 2010.

Detecting nanobubble formation can be accomplished by monitoring the acoustic signature or the scattering of light from the bubbles. Figure 11A shows the photo-acoustic signal of pressure fluctuations in HGN solutions triggered by a NIR laser pulse at time zero [51]. The pressure fluctuations in the solution die out in about 400 µsecs, consistent with the formation of rapidly expanding and collapsing nanobubbles. A more quantitative method of detecting the nanobubbles can be achieved by measuring the scatter of a probe laser by the large refractive index difference between liquid and vapor following an NIR light pulse. A continuous probe laser is aligned collinearly with the NIR pulsed laser, and the transmitted light is directed onto a high speed photodetector and recorded by an oscilloscope (Figure 11B) [120]. Bubble generation results in less light reaching the photodetector from the probe laser due to scattering from the refractive index differences between the nanobubbles and the surrounding liquid. Fast oscilloscope recordings of the photodetector signal (Figure 11B) show a dip in the photodiode signal as nanobubbles form. The magnitude of the dip is proportional to the size and quantity of nanobubbles formed [8,45,48,85,121]. Figure 11B shows that for 20 nm HGN irradiated at 800 nm (the LSPR peak for these HGN was $\lambda_{max}\;$ ~780 nm), bubbles form for fluences ≥ 8 mJ/cm^2^, (red trace) and the amount of light scattered increases with increasing laser fluence, consistent with larger and longer lasting nanobubbles [120]. A fluence of ~3 mJ/cm^2^ was not sufficient to induce bubbles, as indicated by the flat trace (black) of the optical signal. The bubble traces are asymmetric; the decay of the nanobubbles is slower than the nanobubble generation [116]. The extended tail of the trace is likely due to thermal disturbance of the liquid refractive index following nanobubble decay [62] or to generation of secondary bubbles at higher fluences.

Figure 11C is an optical micrograph of a liposome containing carboxyfluorescein decorated with HGN adsorbed to a glass cover slip. Figure 11D shows the same liposome 10 nsec after pulsed NIR irradiation. The image shows the formation of an asymmetric liquid–vapor jet visualized by the variations in refractive index around the liposome as shown schematically in Figure 2 [8]. The mechanical forces generated by these liquid–vapor jets are responsible for lysing liposomes or cell membranes, not the temperature increase in the nanoparticles [122]. Figure 11E shows the release of a fluorescent dye from the liposome interior 10 msec after irradiation. Even at the highest fluences needed for liposome contents release, the measured temperature change in the irradiated suspensions was < 0.5 °C. Nanobubble generation is not due to any changes in the average suspension temperature, which are minimal and incapable of generating a vapor. The process of liposome, endosome, and cell membrane rupture and permeation is due to the mechanical deformations induced by the collapse of the nanobubbles, similar to the changes in liposomes induced by sonication [122], as shown schematically in Figure 2. 

**Figure 12:**
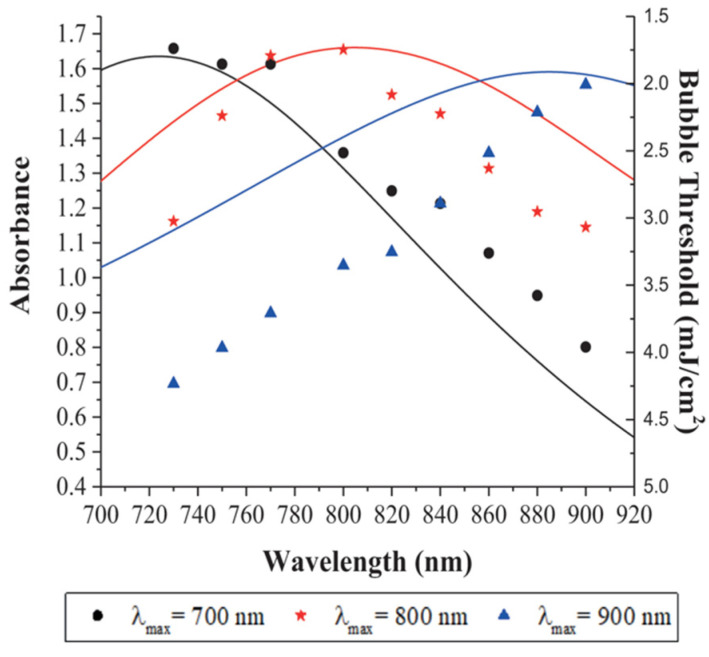
Solid lines show the optical absorption spectra of 20 nm HGN with different wall thicknesses with $\lambda_{max}\;$of 700 nm (black line), 800 nm (red line), and 900 nm (blue line). The nanobubble threshold fluence is smallest when the irradiating wavelength coincides with $\lambda_{max}$ of the HGN. Black circles give the threshold fluence for $\lambda_{max}$ = 700 nm, red stars for $\lambda_{max}$ = 800 nm, blue triangles for $\lambda_{max}$ = 900 nm for different irradiation wavelengths. The minimum threshold fluence is ~ 2 mJ cm^−2^ when irradiated at $\lambda_{max}$ for all HGN. For irradiating wavelengths ≠ $\lambda_{max}$, the fluence to initiate nanobubble generation increases rapidly. Adapted from [45], published by Wiley, 2018.

The amount of energy transferred to the HGN is maximized when the laser wavelength is matched to $\lambda_{max}$ of the HGN, as shown in Figure 12. Three different 30 nm diameter HGN were synthesized to have different $\lambda_{max}$ of 700, 800, and 900 nm (solid lines in Figure 12). The threshold fluence to generate nanobubbles was measured as a function of laser wavelength for the different HGN using the optical scattering method shown in Figure 11B. Figure 12 shows that the minimum fluence to initiate nanobubbles corresponds to the laser frequency that coincides with $\lambda_{max}$ and that the threshold fluence increases as the laser wavelength moves off resonance.

### 2.8. Decreasing Threshold Fluence by Adding Volatile Components to HGN

Shin et al. showed that the hollow cavity of the HGN can be filled with mixtures of perfluoroheptane in tetradecanol (PFC-HGN) to decrease the threshold fluence necessary for nanobubble formation by 50–60% [85]. The spinodal temperature for perfluoroheptane is ~400–430 K, in comparison with that of water of 550 K [117]. The volumetric heat capacity of perfluoroheptane, $\rho_{M}C_{pM}$ = 1.9 × 10^6^ J·m^−3^·K^−1^ [123] is 55% lower than water, $\rho_{M}C_{pM}$ = 4.2 × 10^6^ J·m^−3^·K^−1^. Equation (6) shows that to reach the lower spinodal temperature of perfluoroheptane, the nanoshell only needs to be heat to about 300 °C compared with the ~ 1000 °C needed to induce a nanobubble (Equation (6)). This suggests that the threshold fluence for perfluoroheptane nanobubble formation should be about 30–40% that of water nanobubbles, which is consistent with the observed reduction in nanobubble thresholds of ~60%. Other volatile substances might be encapsulated within the HGN or suspended in liposomes or other carriers to reduce the needed flux. For a given flux, the PFC-HGN generate larger nanobubbles than the HGN, and hence rupture liposomes more efficiently to release more of their contents at a given laser fluence [85]. This may be important for in vivo use where the NIR light is being attenuated by thicker tissue sections [47].

## 3. Liposome Carriers

### 3.1. Conventional Liposomes by Thin Film Hydration and Extrusion

Almost any liposome can be coupled to gold nanostructures. “Conventional” liposomes can be prepared by thin-film hydration, with the desired buffer containing any small molecule dyes or bioactive material to be encapsulated [45,48,124,125]. The desired lipids, cholesterol, PEG-lipids, or PEG-thiol lipids to make up the liposome membrane are mixed in chloroform, and the solvent is evaporated under vacuum overnight. If desired, lipophilic dyes can be added to the lipids in chloroform to label the liposome bilayers. After the solvent is completely removed, the dried lipids are hydrated for 30–60 min at a temperature 10–20 °C above the highest phase transition temperature of the lipids (for dipalmitoylphosphatidylcholine (DPPC), the phase transition temperature is ~45 °C [45,48,125]). The multilamellar aggregates produced by hydration are converted to unilamellar liposomes by extrusion to the desired final size. Prior to extrusion, five or more freeze–thaw cycles (freezing in liquid N_2_ and increasing the temperature to 60 °C) can be carried out if the extrusion does not produce the desired unilamellar liposomes.

To tether HGN to the exterior of the liposome membrane, HGN are mixed with liposomes with thiol-PEG in the membrane overnight at room temperature at relative concentrations to provide 1–10 HGN per liposome. A single HGN on a liposome is sufficient for generating nanobubbles and lysing the liposome. Untethered HGN and unencapsulated cargo materials can be removed by size-exclusion chromatography. The mean size of the liposomes depends on the filter size used during extrusion, and particle tracking and cryo-TEM imaging show that the HGN–liposomes are stable against aggregation or degradation over the course of 2 months [48].

### 3.2. Interdigitation-Fusion Liposomes

**Figure 13:**
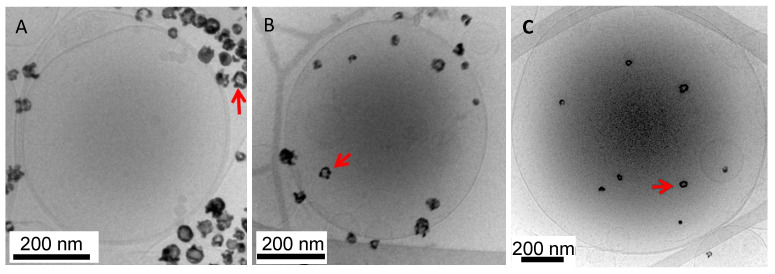
Cryo-TEM images of interdigitated-fusion DPPC liposomes (**A**) mixed with HGN, (**B**) tethered to the HGN via a lipid-PEG-thiol linker, or (**C**) encapsulated within the liposome [51,52].Adapted from [51,52], published by ACS, 2008 and Elsevier, 2009.

To encapsulate HGN or other colloidal sized objects within a liposome, the interdigitated-fusion method can be used. DPPC and other saturated phospholipids form an interdigitated phase on addition of ethanol at temperatures below the main phase transition [124,126]. The bilayers in the interdigitated phase are quite rigid and form flat sheets below the main transition temperature but soften and close into liposomes when the temperature is increased above the main transition temperature [124,127]. First, small unilamellar liposomes of DPPC are prepared by the conventional hydration-extrusion process outlined above. The DPPC (or modified DPPC) liposomes are transformed into interdigitated bilayer sheets by dropwise addition of ethanol (3 molar net ethanol concentration) to the liposome suspension at room temperature [124,127]. The interdigitated sheets are centrifuged at low speed to pellet the sheets and then washed with buffer to remove the ethanol. Following the final wash step, the desired buffer containing any small molecule dyes or bioactive material to be encapsulated is added along with the desired concentration of HGN or other nanoparticles [32,33,126,127]. The solution is held at 55 °C for 20 min to melt the interdigitated phase into the L_α_ or liquid crystalline phase. The bilayers soften, which allows the bilayers to close up into liposomes and encapsulate any nanoparticles, dyes, macromolecules, or other smaller vesicles [125,127]. Liposomes can be separated from unencapsulated nanoparticles by repeated slow-speed centrifugation followed by exchange of the supernatant with fresh buffer. To sterically stabilize the liposomes, a micellar solution of DSPE-PEG of various molecular weights or thiolated DSPE-PEG is added to the liposome solution at 5 mol% of the total liposome lipid concentration at 55 °C, and the mixture is allowed to equilibrate for 24 h. The DSPE-PEG inserts itself into the bilayer of the liposomes [32]. Excess DSPE-PEG can be removed by centrifugation and repeated washing with buffer. TEM images of the interdigitated-fusion liposomes are shown in Figure 13.

### 3.3. Laser Triggering of Contents Release from Liposomes and Other Carriers

The first demonstration of rapid release from dipalmitoylphosphatidylcholine (DPPC) liposome carriers was by Wu et al. [51]. The HGN were encapsulated within the liposome using the interdigitation-fusion process, tethered to the liposomes using a lipid-PEG-thiol linker, or mixed as a liposome–HGN suspension (Figure 13) [51]. The liposomes contained carboxyfluorescein (CF) at a sufficient concentration that CF’s fluorescence was self-quenched prior to release from the liposomes. Disruption of the three sets of liposomes was triggered by irradiation with NIR pulses (*λ*_0_ = 800 nm, 130 fs duration, 1 kHz repetition rate, energy up to 670 μJ/pulse). Irradiation with the pulsed-NIR laser above the threshold power triggered a near instantaneous increase in fluorescence in the liposome solutions containing HGNs but had no effect on the control solutions of liposomes with CF but no HGNs, or on a mixture of HGNs and CF, as shown in Table 1 [52]. Fractional release is taken relative to the solution concentration measured following complete lysis of the liposomes with Triton-X:

(7) $$\%\;Release=\frac{I_{r}-I_{0}}{I_{T}-I_{0}}\times 100\%\;\;\;\;\;\;\;\;\;\;\;\;\;\;\;\;\;\;\;\;\;$$

in which *I_r_* is the measured signal intensity following NIR irradiation, *I*_0_ is the background signal intensity, and *I_T_* is the maximum signal intensity following liposome lysis by Triton X-100.

Table 1 shows that proximity to the liposome membrane determines the fractional release of CF following irradiation. Placing the HGN directly in contact with the liposome membrane via tethering led to near complete disruption of the liposomes. Encapsulating the liposomes also showed a high degree of disruption and CF release. However, simply mixing the liposomes with HGN led to a lower level of CF release. These results are consistent with liposome rupture by the mechanical forces of nanobubble expansion and collapse, as shown in Figure 2 and Figure 11.

**Figure 14:**
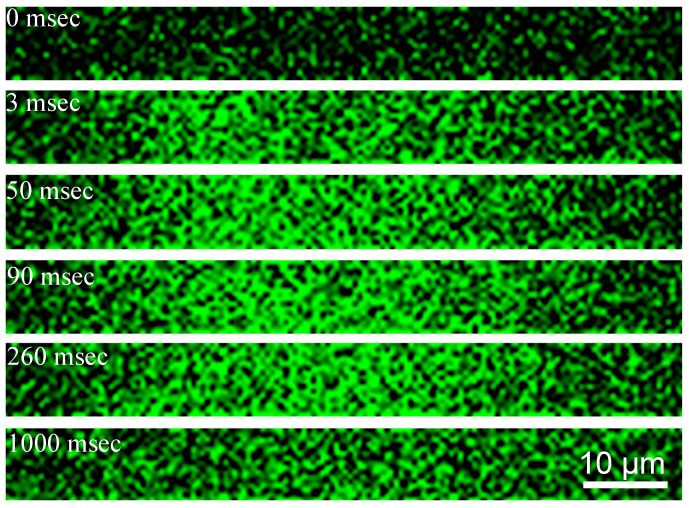
Fluorescence images of release of calcein dye from a suspension of gold-coated liposomes following a single 50 µm diameter light pulse. Prior to irradiation, the calcein fluorescence is quenched in the liposomes. Three microseconds after irradiation, the fluorescence intensity increases and reaches a maximum at ninety seconds. The fluorescence gradually decreases due to dilution. Adapted from [47], published by Wiley, 2020.

Figure 14 shows the release of calcein dye from liposomes coated with gold nanoparticles grown on the liposome surface as in Figure 8 [46,47,128]. A drop of calcein-loaded gold-coated liposome suspension was placed on a glass slide, covered by a cover slide, and sealed. The samples were placed onto a microscope (Olympus IX73) stage and irradiated with a single 740 nm wavelength (matched to the $\lambda_{max}$ of the gold-coated liposomes) picosecond laser pulse of fluence 40 mJ/cm^2^. Prior to irradiation the calcein fluorescence was low due to the self-quenching properties of calcein at high concentration. Following the laser pulse, the fluorescence intensity increased within 3 msec as the calcein was released from the liposomes. At about 90 msec, the calcein fluorescence reached a maximum and then decreased due to diffusion and dilution. This result showed that only a single laser pulse was sufficient for inducing nanobubble formation, liposome rupture, and contents release [47].

### 3.4. In Vitro Release of Bioactive Compounds from Liposomes with NIR light

Bioactive molecules such as calcium, inositol 1,4,5-trisphosphate (IP3), adenosine triphosphate (ATP), and other small, water-soluble “messenger” molecules induce a number of physiological functions. To investigate these processes requires the delivery of a pulse of a particular biologically active molecule at a defined time and place [129,130,131], which led to the synthesis of a variety of photoactivated “caged compounds”. For example, in “caged ATP” [132], the ATP is rendered inactive by covalently bonding a photolabile protecting group to prevent the ATP from reacting. To uncage the bioactive molecule, a pulse of UV light is applied to cleave the covalent bond connecting the protecting group, rendering the molecule biologically active. A variety of neurotransmitters, peptides, and enzymes have been “caged” in a similar fashion by covalent linkages to ultraviolet light-triggered chromophores [129]. The major limitation of these caged compounds is that UV light is rapidly adsorbed by tissue and cells over a few microns and that the UV light can be toxic and degrade proteins and DNA. 

To address these limitations, Webb and Tsien [133] developed new chromophores that could be triggered using two-photon excitation with near-infrared light to uncage compounds. However, covalent bonds still require the equivalent energy of a UV photon to break and uncage the ATP, enzyme, etc. To achieve the equivalent energy to break the covalent bond attaching the photolabile protective group from the bioactive molecule, two-photon excitation requires the simultaneous absorption of two NIR photons of approximately half the energy of the UV photon. This is only possible at the focal point of the two-photon microscope, where the photon density is at a maximum, although still with relatively low probability. The commercialization of two-photon microscopes [134] makes delivering NIR light with cell level resolution accessible to most labs [61,135,136]. The major practical drawback is that each caged compound requires a separate synthesis with an appropriate photolabile caging group tuned to the NIR window [129]. Inorganic ions such as calcium or magnesium cannot be caged but can be trapped in photolabile chelators such as BAPTA, EDTA, and EGTA. Photolysis decreases the chelator ion affinity, releasing some fraction of the chelated ion [137,138].

However, NIR-triggered gold liposomes can deliver almost any water-soluble bioactive molecules, including calcium, ATP, and IP_3_, or water-soluble fluorescent dyes such as calcein or carboxyfluorescein [45,46,47,48]. The liposome carrier is universal and sequesters almost any water-soluble cargo of interest, protects that cargo against degradation, and minimizes premature release. Liposome encapsulated ATP is 85% active compared after 25 min of exposure to ATPase; free ATP is completely degraded [139]. Ion and polar molecule release through liposome membranes is slow; calcium can be held in liposomes for over a month without detectable leakage [48].

**Figure 15:**
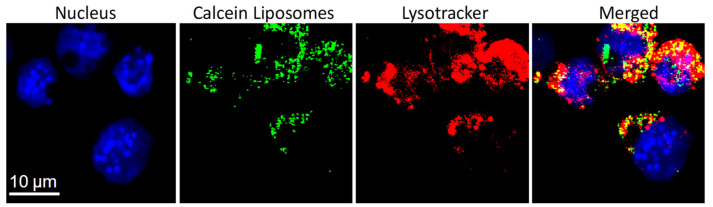
Fluorescence intensity resulting from the intracellular distribution of calcein containing gold-coated liposomes following incubation with RAW 264.7 model macrophage cells. The punctate nature of the calcein fluorescence is consistent with localization of the liposomes in endosomes. This is confirmed by labeling late endosomes and lysosomes with LysoTracker Red DND-99. The merged images shows that the calcein and LysoTracker fluorescence co-localize, confirming that the liposomes are located in the endosomes. Adapted from [47], published by Wiley, 2020.

Liposomes, siRNA [59,60], or functional proteins [61,66,69] can be chemically tethered to HGN and successfully delivered to the cell cytoplasm via endocytosis. Gold-coated liposomes, HGN-tethered liposomes, HGN, and other gold nanoparticles have a negligible effect on cell viability [32,33,47,58,59,60,61,66]. Figure 15 shows calcein containing gold-coated liposomes taken up by endocytosis into Raw 264.7 macrophage model cells. The cells were seeded and cultured in DMEM medium supplemented with 10% fetal bovine serum for 24 h. The cells were then washed with PBS and fresh medium that contained calcein-loaded gold-coated liposomes was added. For the images in Figure 15, 3 h of incubation were allowed for endocytosis of the liposomes. The liposomes co-localized with late endosomes and lysosomes, as shown by the merged fluorescence signals (Figure 15). The punctate distribution of the calcein fluorescence prior to NIR-triggered release is consistent with localization in the endosomes [58].

**Figure 16:**
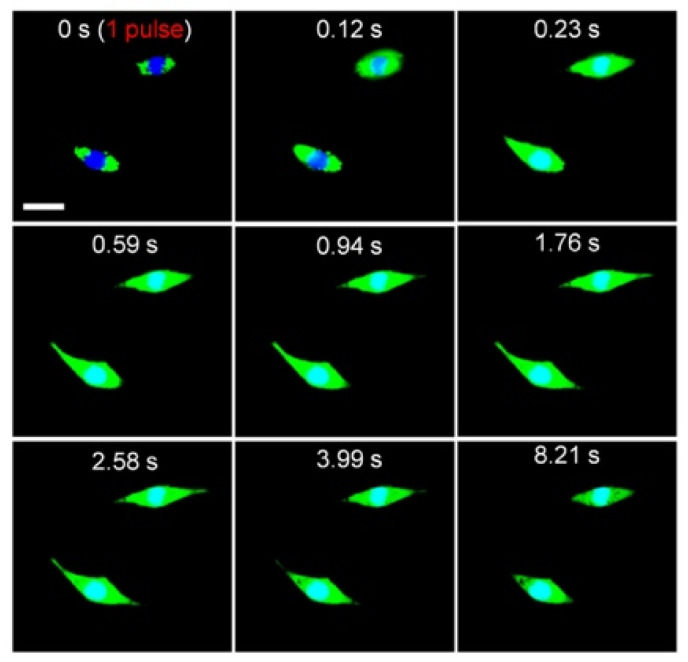
Calcein fluorescence intensity distribution following a single NIR laser pulse. Gold-coated liposomes encapsulating calcein were endocytosed (Figure 15) into RAW 264.7 cells and the endosomes and liposomes ruptured by a single NIR light pulse. The rapid spreading of the calcein dye is indicative of efficient endosome escape. The cells show minimal toxicity for the appropriate NIR fluence, although higher fluences can be used to kill the cells. Bar is 10 µm, and the magnification is the same in all images. Adapted from [47], published by Wiley, 2020.

However, functional molecules can only have an effect if the liposome contents can be released from the endosomes into the cytoplasm. Endosome escape is the main bottleneck of all non-viral vectors; recent reports suggest that only 0.01–2% of endocytosed non-viral vectors reach the cytoplasm [140,141,142,143]. The less efficient the endosome escape, the higher the carrier and cargo dose that is required, which may increase toxicity [142,144]. To escape this “endosome abyss” [143], NIR light-generated nanobubbles can be used to mechanically rupture endosomes and endocytosed liposomes to release their contents [45,51,52,55,85], releasing siRNA [58,59,60] complex functional proteins [61,66,69] and small molecules from liposomes to the cytoplasm [46,47,51,52,120]. Figure 16 shows the NIR light-triggered release of calcein from liposomes endocytosed into RAW 264.7 cells (Figure 15) with a single laser light pulse. The green fluorescence of the calcein spreads throughout the cell over a few seconds, eclipsing the blue fluorescence in the cell nucleus (Figure 15). The nanobubbles induced by the NIR light pulse ruptured not only the liposomes containing the calcein but also the endosomes, resulting in the release of calcein to the cytoplasm and the spread of calcein fluorescence throughout the cell. For the proper choice of fluence, no toxicity is observed, although higher fluences can be used to lyse the cells.

**Figure 17:**
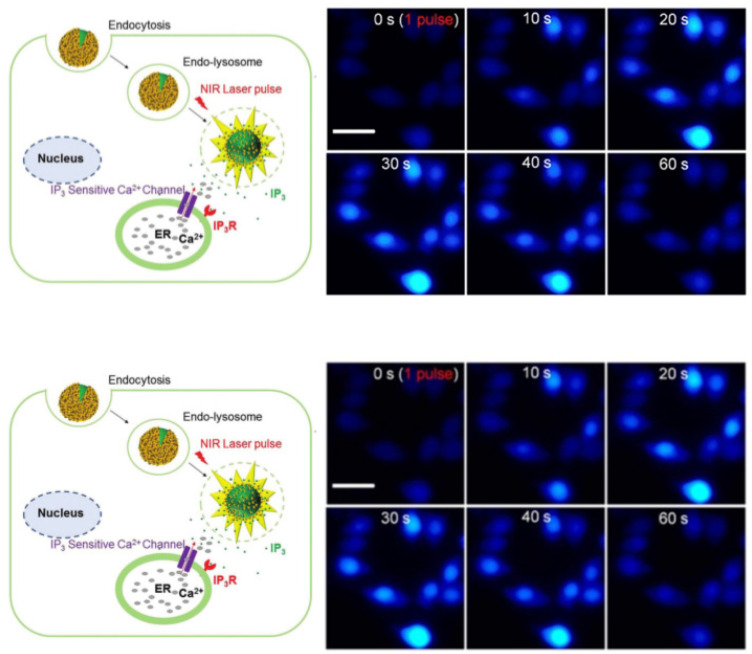
(**Left**): Schematic of gold-coated liposomes containing IP_3_ entering the cell via endocytosis. A single laser pulse induces vapor nanobubbles that rupture the liposome- and endosome-releasing IP_3_ to the cytoplasm. The IP_3_ opens calcium channels, thereby increasing the calcium concentration in the cytoplasm detected by the Fluo-4 calcium indicator. (**Right**): Time sequence of Fluo-4 fluorescence in RAW 264.7 cells following NIR light-induced rupture of IP_3_-containing liposomes trapped in endosomes. The blue fluorescence signal within each cell increases up to about 30 sec, indicating release of calcium from the ER. Bar is 10 µm, and the magnification is the same in all images. Adapted from [46,47], published by Wiley, 2017 and Wiley, 2020.

Figure 17 shows that rapid endosome escape via NIR light pulses can release second messenger molecules and trigger complex intracellular functions. Inositol 1,4,5-trisphosphate (IP3) is a second messenger for intracellular calcium ion signaling [47]. IP_3_ was encapsulated within gold-coated liposomes and incubated with RAW 264.7 cells, as in Figure 15, leading to endocytosis. IP_3_ in the cytosol binds to a receptor located on the endoplasmic reticulum membrane, which causes calcium release from the endoplasmic reticulum into the cytosol [145]. A single pulse of NIR light releases the IP_3_ from the liposomes and endosomes and causes a transient increase in the intracellular calcium concentration, as shown by the increase in Fluo-4 fluorescence.

Nanobubble-triggered endosome escape makes siRNA delivery possible at an order of magnitude lower dose than lipofectamine [60,146] and can be used to pattern 3D human embryonic stem cell spheroids [136]. Two-photon microscopes can be used to deliver the NIR light pulses with sub-cellular resolution [61], or cells and liposomes can be triggered in microfluidic flow using stand-alone lasers [66]. Functional, high molecular weight proteins such as Cre Recombinase or green fluorescent protein can be delivered via plasmonic gold nanoparticles without protein degradation [61,66,69]. Following endosome rupture, gold nanoparticles are rapidly passivated by endogenous glutathione bonding via the cysteine thiol [147] and are then eliminated from the cell. In addition to enhancing delivery, NIR-triggered release can be patterned at high resolution using the NIR light from a two-photon microscope [58,61].

### 3.5. Differential Release from Different Liposome/HGN Combinations

**Figure 18:**
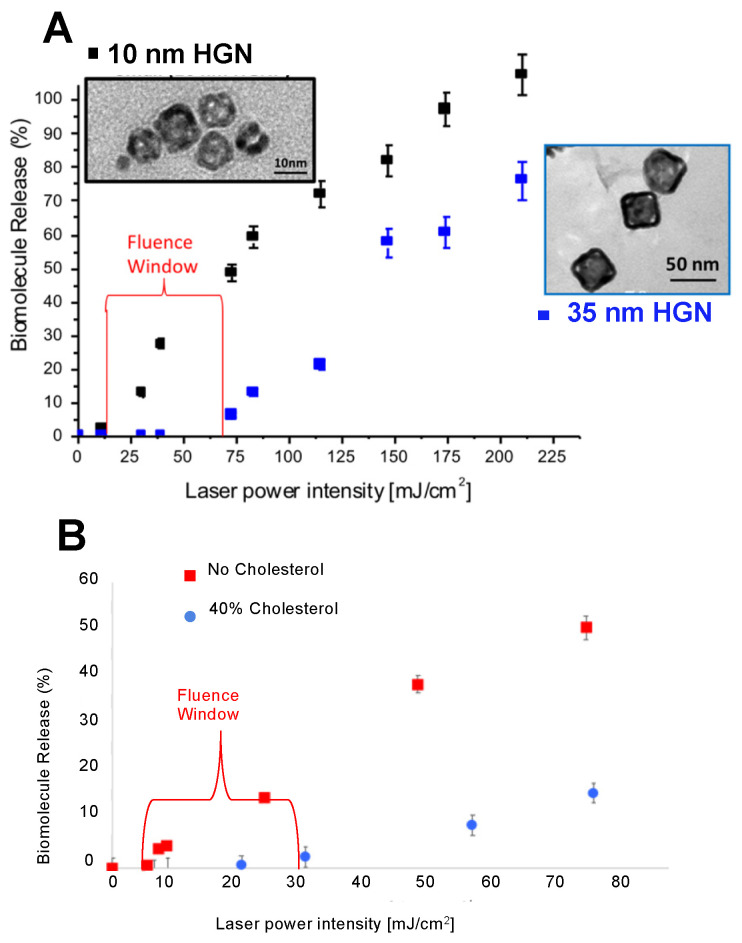
(**A**) Liposomes tethered to different HGN with different light adsorption properties induce calcium release at different laser fluences when irradiated with 800 nm NIR light. This creates a “fluence window” over which one liposome–HGN releases its contents while the second remains intact. (**B**) A fluence window is opened between liposomes with identical HGN but different liposome membrane compositions. Adding cholesterol to DPPC liposomes increases the membrane lysis tension, which increases the laser fluence needed to release calcium. Adapted from [48], published by Nature, 2020.

As shown in Figure 12, the threshold fluence for nanobubble generation depends on the plasmon resonance wavelength, $\lambda_{max}$ relative to the laser wavelength, and the magnitude of the absorption cross section, $\;\sigma_{abs}$. The threshold also depends on the HGN size and/or shell thicknesses, which determine the maximum temperature that the nanoshell and the liquid trapped in the hollow core might reach (Equation (6)) (Figure 9). The liposome membrane composition can also be adjusted to maximize [48] or minimize [47] the lysis tension and mechanical integrity of the liposome. As a result, by tethering HGN of various sizes and $\lambda_{max}$ to liposomes of different composition, it is possible to release one set of liposome contents with light of a given wavelength at a given fluence, then release the contents of a second batch of liposomes at a different wavelength and/or fluence [48]. This “fluence window” is the difference in laser fluence needed to release the contents from one type of HGN–liposome without inducing contents release from a second type of HGN–liposome. Figure 18A shows an ~50 mJ/cm^2^ fluence window for calcium release from liposomes following NIR light for liposomes with 10 nm vs. 35 nm cubic HGN (Figure 18A) with different $\lambda_{max}$ and $\sigma_{abs}$. Figure 18B shows that liposomes tethered to identical HGN but made from lower lysis tension DPPC vs. higher lysis tension mixtures of DPPC and cholesterol can generate a ~20 mJ/cm^2^ fluence window.

#### Controlling ATP to Calcium Ratio by Triggering Mixed Liposomes

With fluence windows made by controlling HGN size, $\lambda_{max}$ and $\sigma_{abs}$, irradiation wavelength, and liposome composition, the sequence or time release of two or more populations of liposome–HGNs can be achieved in the same solution. Due to the universal nature of liposome contents release, any small molecule cargo release only depends on the properties of the liposomes and/or the tethered HGN and the irradiating wavelength. The order of contents release can be inverted by simply changing the HGN–liposome carriers, showing the flexibility of the HGN–liposome delivery platform, especially in comparison with traditional caged compounds.

**Figure 19:**
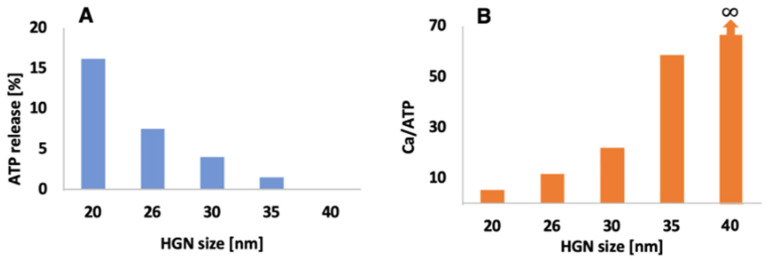
(**A**) Liposomes containing calcium were tethered to 10 nm HGN and mixed with equal volumes of liposomes with 20 nm, 26 nm, 30 nm, 35 nm, and 40 nm HGN encapsulating ATP. All HGN had an identical $\lambda_{max}$ of 800 nm. The mixed liposomes were triggered with single pulses of 800 nm NIR light at a constant fluence of 42 $\mathrm{mJ}/{\mathrm{cm}}^{2}$. This fluence was sufficient to induce ~90% calcium release. However, the fraction of ATP released from otherwise identical liposomes decreased with increasing HGN size, dropping to 0 for 40 nm HGN. (**B**) This gives a release ratio of calcium to ATP that could be varied from 5:1 for the 20 nm HGN to infinity for the 40 nm HGN. Adapted from [48], published by Nature, 2020. By controlling the liposome and HGN properties, any ratio of liposome contents can be released from a mixture of liposomes simultaneously with a single laser pulse. Adapted from [48], published by Nature, 2020.

Figure 19A shows the flexibility of HGN–liposome release. Calcium-containing liposomes were tethered to 10 nm HGN, while ATP containing liposomes were tethered to 20 nm HGN, 26 nm HGN, 30 nm HGN, 35 nm HGN, and 40 nm HGN. The $\lambda_{max}$ of all the HGN were 800 nm, and 800 nm NIR pulses at 42 mJ/cm^2^ were used to trigger contents release. A mixture was made from equal volumes of calcium-containing HGN–liposomes and ATP containing HGN–liposomes. The 10 nm HGN calcium-containing liposomes had a constant ~90% calcium release in all mixtures. However, as shown in Figure 19A, ATP release decreased significantly with increasing HGN size—for 40 nm HGN, the ATP release dropped to 0%. This provided an increase in the calcium to ATP release ratio of 5, 12, 22, 58, and ∞  for 20 nm HGN, 26 nm HGN, 30 nm HGN, 35 nm HGN, and 40 nm HGN, respectively (Figure 19B) [48].

### 4. Co-Delivery of Antibody Labelled Liposomes and Gold Nanoparticles

**Figure 20:**
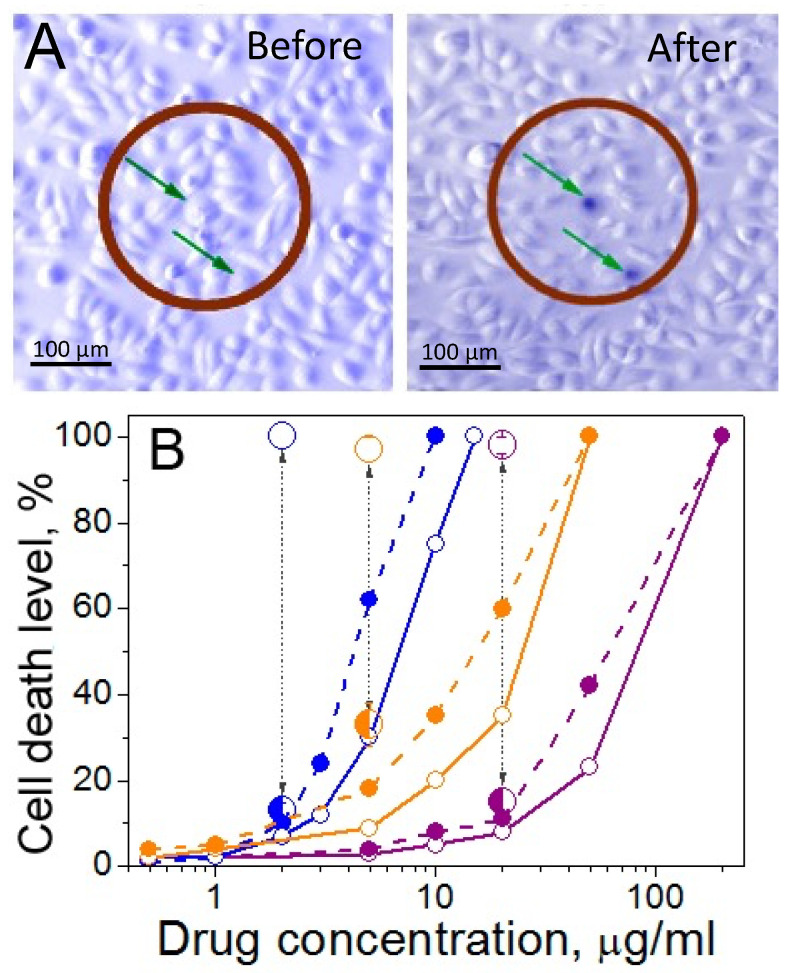
(**A**) **Before**: Bright-field optical microscopy images of a co-culture of normal (NOM9) and squamous cell carcinoma cells (HN31, green arrows) incubated with gold nanoparticles labeled with EFGR antibodies. **After:** A single laser pulse (70 psec, 820 nm, 40 mJ cm^−2^) induced vapor nanobubbles in the HN31 cells without damaging the surrounding normal cells. The dead HN31 cells were stained with Trypan Blue (blue—dead cells, white—live cells). (**B**) Cell death level among cancer (solid lines) and normal (dotted lines) cells measured after 72 h of continuous free doxorubicin (blue), free cisplatin (orange), and liposome-encapsulated doxorubicin (Doxil, purple) exposure as a function of drug concentration. HN31 cells are drug-resistant; cancer cell death levels were lower than normal cells at all drug concentrations. NIR light-induced nanobubble formation induced by endocytosed antibody-labelled HGN increased the death level for cancer cells by 25 times for cisplatin, 6 times for doxorubicin, and 33 times for Doxil, while the death level remained low among normal cells (large circles). Near 100% cancer cell death was achieved at 10-fold reduced drug concentrations. Adapted from [119], published by Oxford, 1989.

Superficial tumors such as oral cavity squamous cell carcinoma (OCSCC) are particularly accessible for topical application of gold nanoparticles and liposomal drugs with targeting and activation by near-infrared light. Both gold nanoparticles and liposomes can be selectively targeted to tumor cells using antibodies to overexpressed surface ligands. Figure 20A shows a co-culture of normal (NOM9) and squamous cell carcinoma (HN31 arrows) following 24 h of incubation with gold nanoshells conjugated to panitumumab antibodies targeting the HN31 cancer cells that overexpress epidermal growth factor receptor (EGFR). The antibody-enhanced endocytosis occurred primarily in the HN31 cells, resulting in nanoparticle clusters localized in the endosomes. These NP clusters in antibody-targeted cells can nucleate vapor nanobubbles at lower fluence than in normal cells that may have endocytosed individual gold nanoparticles or no nanoparticles. The cells within the brown circles in Figure 20A were irradiated with a 260 µm diameter laser beam of wavelength 820 nm at a fluence of 40 mJ/cm^2^. Vapor nanobubbles resulting in cell death occurred primarily in the tumor cells targeted by the antibody, as indicated by the dark blue staining with Trypan Blue. The difference in threshold fluence between the clustered gold nanoparticles and isolated nanoparticles amplifies the targeting of the tumor cells. Most normal cells survived this treatment, even those adjacent to cancer cells in which nanobubbles were generated, showing the extremely localized effects of nanobubbles on cell integrity.

Figure 20B shows the effects of combining chemotherapy agents with NIR light-induced nanobubbles. Free cisplatin and doxorubicin and liposome-encapsulated doxorubicin (Doxil) were added to the co-cultures in (A) prior to triggering nanobubble generation with NIR light. Figure 20B shows the death level among cancer and normal cells measured after 72 h of continuous drug exposure as a function of drug concentration. HN31 cells are drug-resistant; cancer cell death levels (solid lines) were lower than normal cells (dotted lines) at all drug concentrations. However, following NIR light-induced nanobubble formation, compared with the drugs or with nanobubbles alone, the death level for cancer cells increased by 25 times for cisplatin, 6 times for doxorubicin, and 33 times for Doxil, while the death level remained low among normal cells. Near 100% cancer cell death was achieved at 10-fold reduced drug concentrations (Figure 20B); similar cell death levels for the drugs alone required an order of magnitude higher drug dose. Reduced drug doses together with a lower laser fluence required to initiate effective PNBs in cancer cells resulted in a significant reduction in non-specific toxicity for the combination treatment (large circles in Figure 20B).

Lukianova-Hleb et al. [64,65] used this strategy for intra-operative and preventive treatment of head and neck squamous cell carcinoma in mouse models. Prior to surgical resection, gold nanoparticles and liposome-encapsulated chemotherapeutics are administered locally or systemically. Both gold nanoparticles and liposomes have attached antibodies to recognize ligands overexpressed on the tumors. This, combined with the enhanced permeation and retention effect, allows both the nanoparticles and chemotherapeutic liposomes to accumulate preferentially in the tumor. Following surgical resection of the bulk tumor, the tumor margins were exposed to NIR laser pulses via an endoscope. As shown in Figure 20, the laser pulses generated nanobubbles that would preferentially destroy any residual cancer cells at the tumor margins. The laser treatment could be followed up with targeted X-rays that are also enhanced by interaction with the gold nanoparticles in the tumor cells. Local recurrence after 2–4 weeks was 100% for surgery alone, while surgery accompanied by NIR-triggered nanobubble release of chemotherapeutics and X-ray therapy eliminated tumor recurrence [64,65]. 

## 5. Summary

HGN–liposomes or gold-coated liposomes that can be triggered by nanosecond pulses of near-infrared light can provide spatially and temporally localized delivery of many different cargoes in vitro, ex vivo, and in vivo. This combined liposome–plasmon-resonant nanoparticle delivery platform separates the delivery mechanism from the method of sequestering the cargo to be delivered. The delivery platform is also generic in that almost any water-soluble molecule that can be encapsulated in a liposome will be delivered in the same way, in comparison with “caged compounds” that require the chemical synthesis of an individual “cage” with different chemical and physical properties. Liposome cargo release is activated by nanosecond pulses of NIR light that can be delivered by two-photon microscopes, by stand-alone lasers in well plates or during microfluidic flow, or via endoscopes in vivo. NIR light can penetrate millimeters to centimeters in cell suspensions and tissue [45,64]; UV or visible light cannot penetrate more than 10–20 microns. Release is accomplished by the generation of unstable vapor nanobubbles; their violent collapse leads to mechanical forces that rupture liposomes and cell membranes. However, functional proteins and complex oligonucleotides are not degraded during this process and can induce physiological changes following release to the cell cytoplasm. All liposome cargoes—including calcium, ATP, IP_3_, doxorubicin, cisplatin, calcein, and carboxyfluorescein (CF)—can be released from different liposome–HGN combinations by controlling the HGN size and the liposome membrane composition and by irradiating on or off resonance. As a result, different liposomes with different cargoes can be triggered at different times and locations. The combination of unilamellar liposomes, with their ability to encapsulate almost any water-soluble molecule, and plasmonic gold nanostructures, which can be nucleate vapor nanobubbles following a threshold fluence of near-infrared light, provides a unique and universal method of *in vitro* and *in vivo* delivery with spatial and temporal control.

## Figures and Tables

**Table 1:** Percentage (%) release from liposomes.

Laser	Solution	Release (%)
Pulsed	CF + HGN	1 ± 2
Pulsed	Liposomes with CF, no HGN	0 ± 2
Pulsed	Liposomes with CF mixed with HG	28 ± 2
Pulsed	HGN and CF encapsulated in liposomes	71 ± 1
Pulsed	HGN tethered to liposome with CF	93 ± 2
Continuous	HGN tethered to liposome with CF	0 ± 2

## Data Availability

All data relevant to the publication are included.
